# The complete chloroplast genome sequence of *Begonia pedatifida*

**DOI:** 10.1080/23802359.2024.2410444

**Published:** 2024-09-30

**Authors:** Huan Li, Tianlei Zhang, Jiale Liang, Xuan Tang, Zhuoyu Chenyang, Rong Zhu, Ying Chen, Qishan Kuang, Rongjie Huang, Hanbin Yin, Xingyu Zeng, Yongle Liu, Zhitian Du, Kerui Huang, Lei Sun

**Affiliations:** a Hunan Provincial Key Laboratory for Molecular Immunity Technology of Aquatic Animal Diseases, College of Life and Environmental Science, Hunan University of Arts and Science, Changde, Hunan, China; b Key Laboratory of Research and Utilization of Ethnomedicinal Plant Resources of Hunan Province, College of Biological and Food Engineering, Huaihua University, Huaihua, China

**Keywords:** *Begonia pedatifida*, phylogenetic analysis, chloroplast genome

## Abstract

*Begonia pedatifida* has persistently been utilized as a traditional folk herbal medicine. This study has sequenced the chloroplast genome of *B. pedatifida* to establish its genomic characteristics and to discern its phylogenetic relationships with other closely related species. The chloroplast genome structure of *B. pedatifida* reveals a circular molecule with a length of 169,606 bp, including a large single copy (LSC) region of 76,086 bp, a small single copy (SSC) region of 18,314 bp, and a pair of inverted repeats (IRS) region of 37,603 bp. The entire genome contains 138 genes, which consist of 88 protein-coding genes, 42 tRNA genes, and 8 rRNA genes. Phylogenetic analysis suggests that *B. pedatifida* is closely related to *Begonia emeiensis*, *Begonia jinyunensis*, and *Begonia pulchrifolia*, sharing a common ancestor and forming sister lineages. This research provides genetic information for further study on *B. pedatifida*.

## Introduction

**Figure 1.**
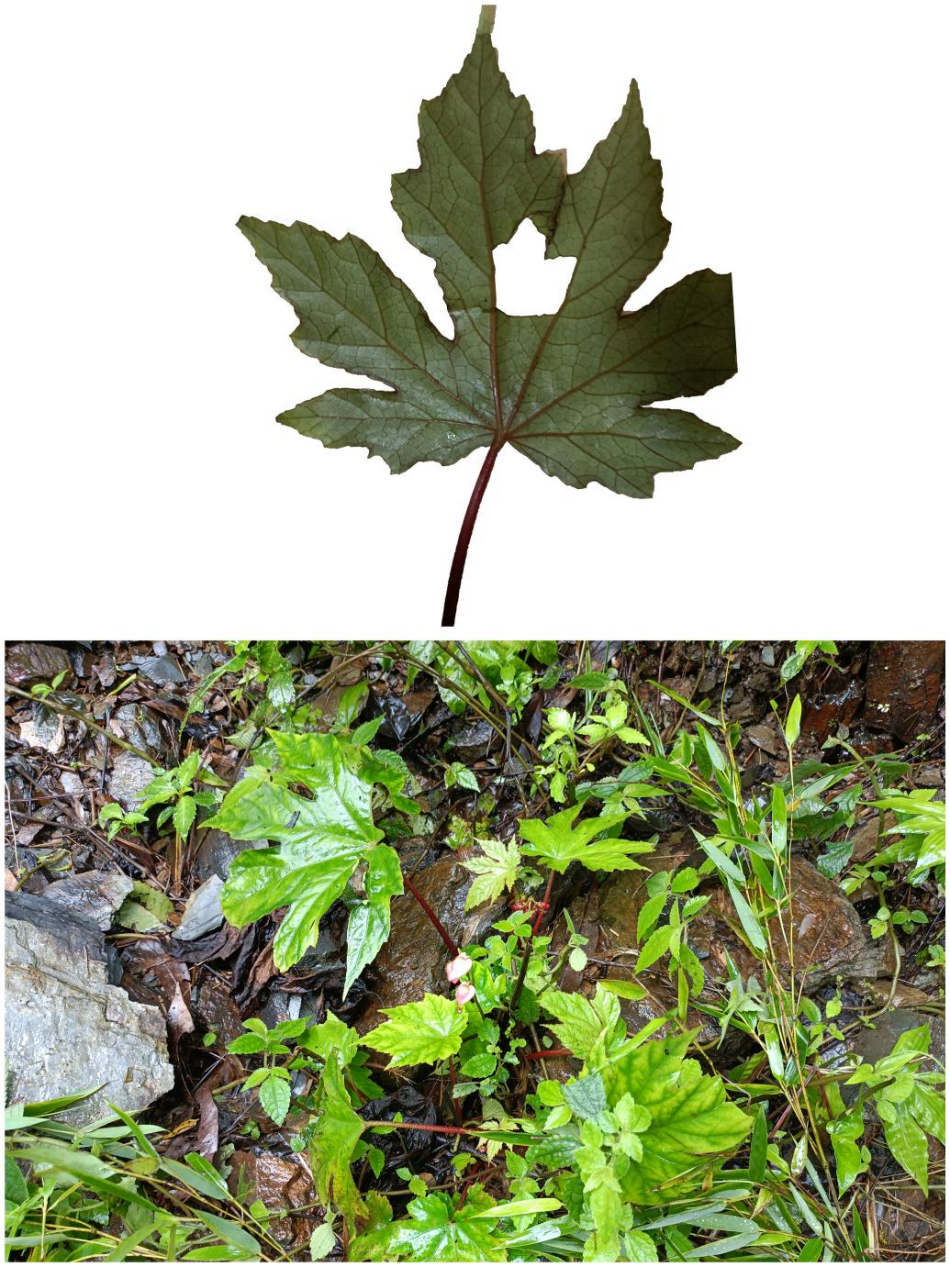
The picture of the collected sample of *Begonia pedatifida*. *Note:* The picture was self-taken by Hanbin Yin near Hengshan in Changde, Hunan Province, China (approximately latitude 27°26′46.3648″ N, longitude 112°43′11.9952″ E, altitude 357 meters). While *B. pedatifida* and *B. circumlobata* share similar characteristics, they can be distinguished by subtle differences. *B. pedatifida* exhibits more complex leaf dissection, with the central lobes further divided, whereas *B. circumlobata* lobes typically remain undivided. Additionally, *B. pedatifida* has triangular leaf margin serrations without awns, in contrast to *B. circumlobata.*

*Begonia pedatifida* is a perennial herbaceous plant of the Begoniaceae family, that grows to a height of 15–40 cm (Figure 1). Its rhizome is light brown and branching, measuring 5–15 cm in length. The leaves are nearly round with deep palmate lobes that are oblong-lanceolate. The flowers have a diameter of 3–3.5 cm, and the inflorescence exhibits a binary cyme structure with pink tepals (Guo 2002).

*Begonia pedatifida* thrives in moist forests or on cliffs and is distributed in Hubei, Hunan, Guizhou, and Sichuan province (Guo 2002). Although considered a weed, *B. pedatifida* possesses ornamental value. Moreover, it possesses various medicinal properties, including significant anti-inflammatory, antioxidant, and anti-tumor effects (Dong et al. 2015). These attributes make it promising in the field of traditional Chinese medicine. The rootstock is used in the treatment of various inflammatory diseases, such as arthritis (Huang et al. 2016). Its extracts contain alkaloids, flavonoids, phenolic compounds, terpenoids, and more. Flavonoids can lead to anticancer effects, so it has shown potential in the treatment of tumors (Prihardina and Fatmawati 2021). The plant’s rich content of antioxidants, including anthocyanins and phenolic compounds, not only aids in scavenging free radicals and safeguarding cells against damage but also contributes to slowing down the aging process. This suggests potential applications in health and anti-aging products (Bhattarai and Rana 2022).

Despite its prevalence, research on *B. pedatifida* is limited, particularly concerning its genome and evolutionary status. The absence of gene sequence and characterization information hinders its value exploitation and necessitates further investigation. This study aims to sequence and characterize the chloroplast genome of *B. pedatifida* to investigate its phylogenetic status and conduct phylogenetic analysis to provide a foundation for further research.

## Materials

For the plant material, fresh leaves were collected from *B. pedatifida*, a species cultivated near Hengshan in Changde, Hunan Province, China (approximately latitude 27°26′46.364″ N, longitude 112°43′11.9952″ E, altitude 357 meters). The voucher specimen is preserved at the College of Life and Environmental Sciences, Hunan University of Arts and Sciences (Contact Person: Kejiang Huang, huangkerui008@163.com, voucher number ZLY002). Due to the morphological similarities between *B. pedatifida* and *B. circumlobata*, we conducted a verification process to confirm the identity of the species collected in this study. Our identification of the specimens as *Begonia pedatifida* was corroborated through both morphological examination (Figure 1) and sequence analysis (Figure S3). For sequence analysis, to identify and compare our collected specimen (OR288087) with *B. pedatifida* and *B. circumlobata*, all available *rpl16* coding sequences and trnL-trnF intergenic spacer regions for *B. pedatifida* and *B. circumlobata* were downloaded from NCBI. Multiple sequence alignments were performed to assess genetic similarities and differences among the taxa. Additionally, we mapped the trnL-trnF sequence of *B. circumlobata* onto the OR288087 chloroplast genome to identify subtle differences.

## Methods

DNA extracts from leaves preserved in liquid nitrogen were obtained using the DN Easy plant tissue kit (TIANGEN Biotech Co., Ltd., Beijing). Subsequently, the library was prepared and sequenced on the Illumina HiSeq 2500 platform (Shanghai personalbio Technology Co., Ltd., China). The results revealed that after filtering out low-quality reads with fastp (Chen et al. 2018) a total of 84,574,200 reads were retained. De novo assembly of the *B. pedatifida* chloroplast genome was carried out using GetOrganelle v1.7.5 (Jin et al. 2020). The chloroplast genome structure is reliable and the sequencing depth distribution at each site is relatively stable in most cases (Figure S1 and S4). CPGAVAS2 (Shi et al. 2019) was utilized for chloroplast genome annotation. Finally, the genomic map was created using CPGView (Liu et al. 2023). Phylogenetic analysis was conducted following these steps: initially, 50 chloroplast genomes were downloaded from GenBank, and 61 protein-coding genes shared among all genomes were identified. Subsequently, MAFFT v7.313 (Rozewicki et al. 2019) was employed for individual gene alignment. Gblocks 0.91b was then used to mask each gene’s sequence, and end-to-end connections were made for all genes to form a supergene for each species (Guo et al. 2022). Maximum likelihood phylogenies were inferred using IQ-TREE (Nguyen et al. 2015) under the TVM+F + I + G4 model automatically selected by IQ-TREE ('Auto’ option in IQ-TREE) for 5000 ultrafast bootstraps, as well as the Shimodaira–Hasegawa–like approximate likelihood-ratio test.

## Results

**Figure 2.**
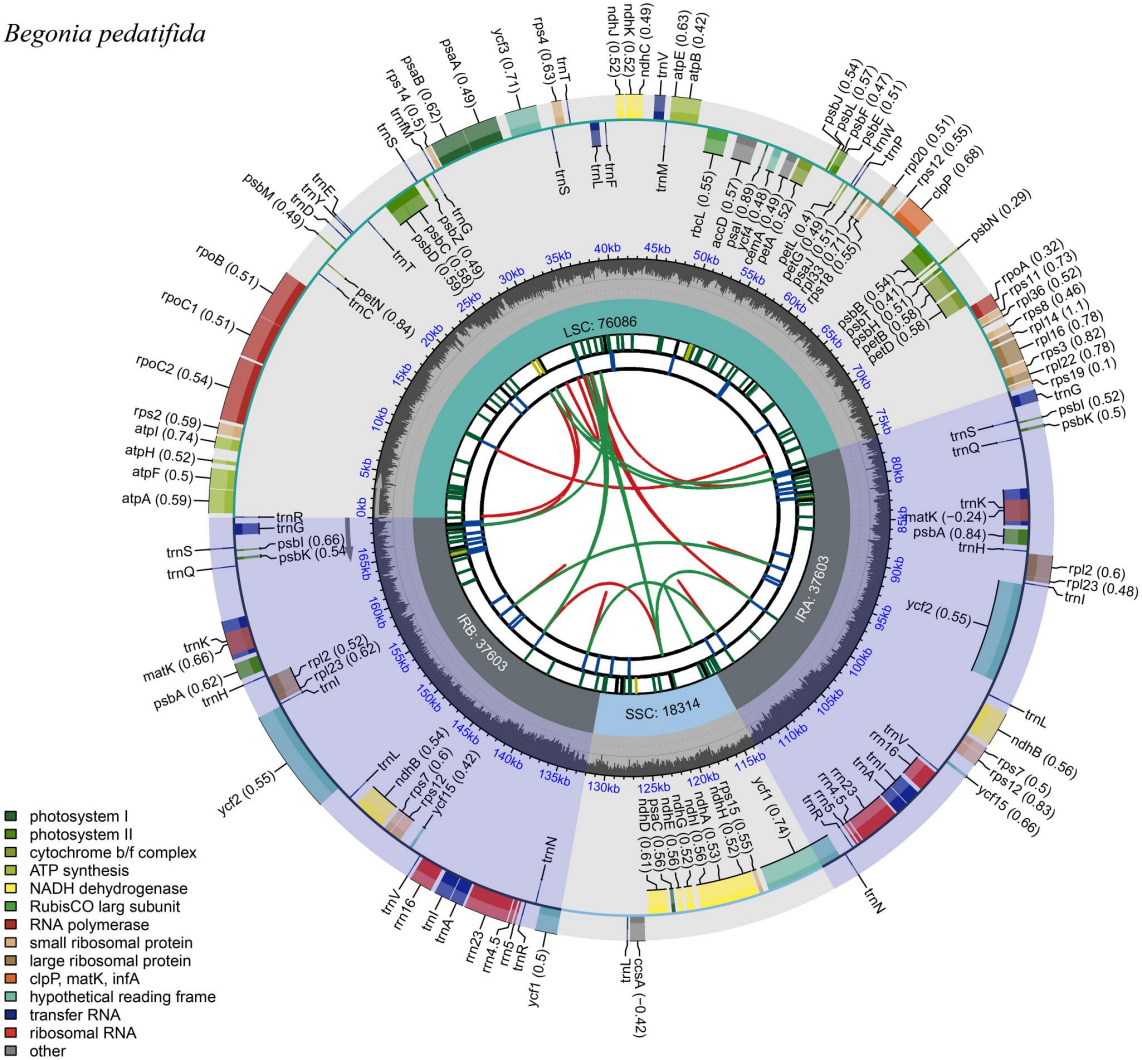
Gene map of the *Begonia pedatifida* chloroplast genome. From the center outward, the first track indicates the dispersed repeats. The second track shows the long tandem repeats as short blue bars. The third track shows the short tandem repeats or microsatellite sequences as short bars with different colors. The fourth track shows small single-copy (SSC), inverted repeat (ira and irb), and large single-copy (LSC) regions. The GC content along the genome is plotted on the fifth track. The genes are shown on the sixth track.

The chloroplast gene structure of *B. pedatifida* is a circular molecule, with a length of 169,606 bp including four parts: a large single-copy region (LSC) length of 76,086 bp, a small single-copy region (SSC) length of 18,314 bp, and two inverted repeat regions (IRs), each 37,603 bp (Figure 2). The G + C content was 35.56% for the whole chloroplast genome, and 38.91% for the IRs, which was higher than that in LSC and SSC regions (33.88% and 28.80%, respectively). The genome contains 138 genes, including 88 protein-coding genes, 42 tRNA genes, and 8 rRNA genes, of which 29 were duplicated in the IR regions. The structure of the cis-splicing genes and trans-splicing genes were shown in Figure S2, there are 12 cis-splicing genes and 1 trans-splicing gene.

**Figure 3.**
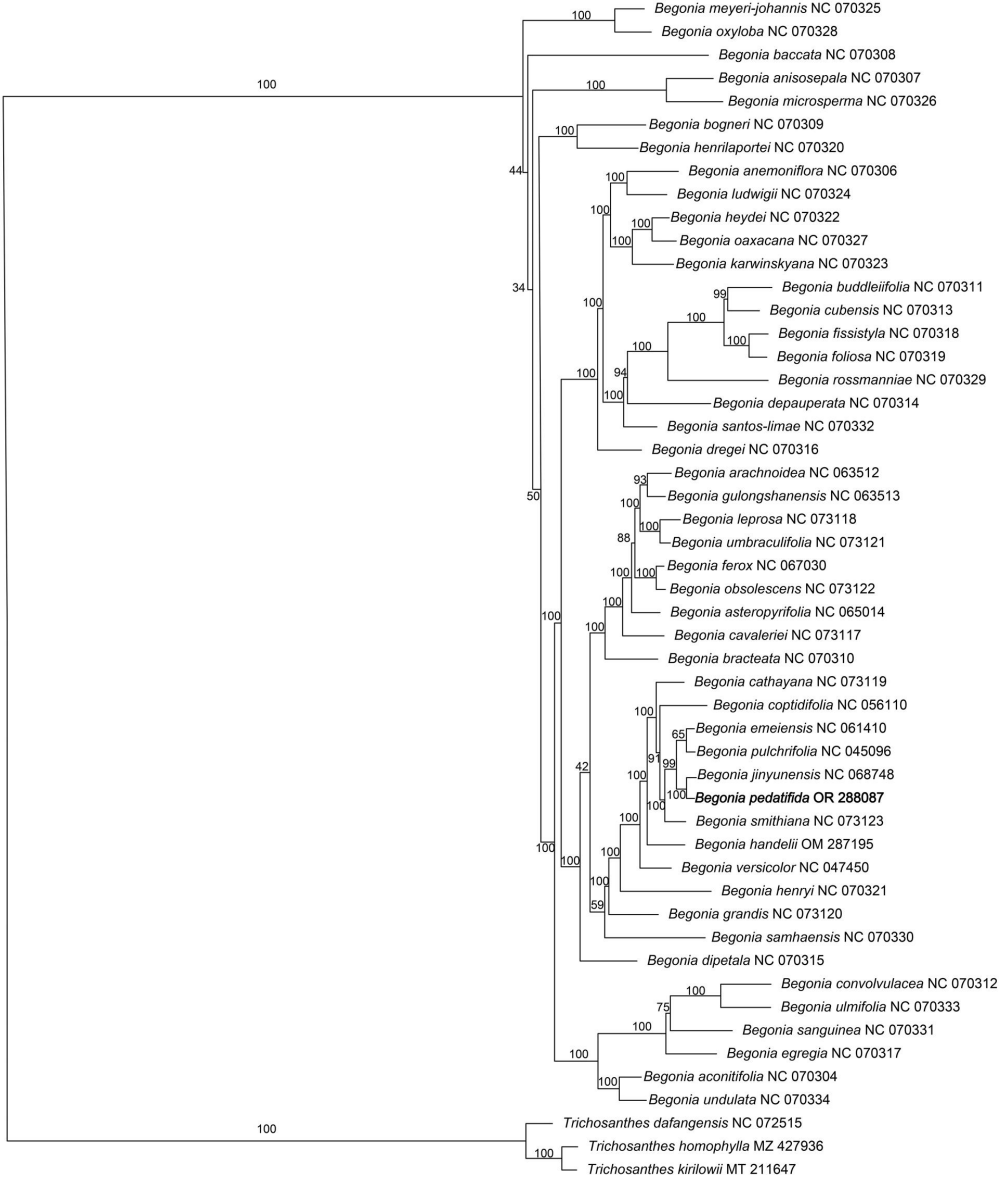
Maximum-likelihood (ML) tree of *Begonia pedatifida* and 50 relative species was reconstructed using the IQ-tree based on 61 protein-coding genes shared by all genomes. Bootstrap values are shown next to the nodes. The following sequences, of which some existed in NCBI database but were unpublished, were used: *Begonia aconitifolia* NC 070304 (Tseng et al. 2022), *Begonia anemoniflora* NC 070306 (Tseng et al. 2022), *Begonia anisosepala* NC 070307 (Tseng et al. 2022), *Begonia arachnoidea* NC 063512 (Tao et al. 2022), *Begonia asteropyrifolia* NC 065014 (Lu and Luo 2023), *Begonia baccata* NC 070308 (Tseng et al. 2022), *Begonia bogneri* NC 070309 (Tseng et al. 2022), *Begonia bracteata* NC 070310 (Tseng et al. 2022), *Begonia buddleiifolia* NC 070311 (Tseng et al. 2022), *Begonia cathayana* NC 073119 (Xiong et al. 2023), *Begonia cavaleriei* NC 073117 (Xiong et al. 2023), *Begonia convolvulacea* NC 070312 (Tseng et al. 2022), *Begonia coptidifolia* NC 056110 (Wang et al. 2021), *Begonia cubensis* NC 070313 (Tseng et al. 2022), *Begonia depauperata* NC 070314 (Tseng et al. 2022), *Begonia dipetala* NC 070315 (Tseng et al. 2022), *Begonia dregei* NC 070316 (Tseng et al. 2022), *Begonia egregia* NC 070317 (Tseng et al. 2022), *Begonia emeiensis* NC 061410、*Begonia ferox* NC 067030 (Zhou et al. 2023), *Begonia fissistyla* NC 070318 (Tseng et al. 2022), *Begonia foliosa* NC 070319 (Tseng et al. 2022), *Begonia grandis* NC 073120 (Xiong et al. 2023), *Begonia gulongshanensis* NC 063513 (Guan et al. 2022), *Begonia handelii* OM287195 (Zhu et al. 2022), *Begonia henrilaportei* NC 070320 (Tseng et al. 2022), *Begonia henryi* NC 070321 (Tseng et al. 2022), *Begonia heydei* NC 070322 (Tseng et al. 2022), *Begonia jinyunensis* NC 068748, *Begonia karwinskyana* NC 070323 (Tseng et al. 2022), *Begonia leprosa* NC 073118 (Xiong et al. 2023), *Begonia ludwigii* NC 070324 (Tseng et al. 2022), *Begonia meyeri-johannis* NC 070325 (Tseng et al. 2022), *Begonia microsperma* NC 070326 (Tseng et al. 2022), *Begonia oaxacana* NC 070327 (Tseng et al. 2022), *Begonia obsolescens* NC 073122 (Xiong et al. 2023), *Begonia oxyloba* NC 070328 (Tseng et al. 2022), *Begonia pulchrifolia* NC 045096 (Fan et al. 2019), *Begonia rossmanniae* NC 070329 (Tseng et al. 2022), *Begonia samhaensis* NC 070330 (Tseng et al. 2022), *Begonia sanguinea* NC 070331 (Tseng et al. 2022), *Begonia santos-limae* NC 070332 (Tseng et al. 2022), *Begonia smithiana* NC 073123 (Xiong et al. 2023), *Begonia ulmifolia* NC 070333 (Tseng et al. 2022), *Begonia umbraculifolia* NC 073121 (Xiong et al. 2023), *Begonia undulata* NC 070334 (Tseng et al. 2022), *Begonia versicolor* NC 047450 (Zhou et al. 2020), *Trichosanthes dafangensis* NC 072515, *Trichosanthes homophylla* MZ427936, *Trichosanthes kirilowii* MT211647 (Yang et al. 2019).

Based on the chloroplast genome of *B. pedatifida*, the Maximum-likelihood (ML) tree was constructed (Figure 3), which shows the phylogenetic placement of *B. pedatifida.* The result shows that *B. pedatifida* is very close to *B. jinyunensis* and *B. pulchrifolia*, sharing a common ancestor, which is consistent with previous studies that used the ITS1, ITS2, and 5.8S gene regions to construct phylogenetic trees (Feng et al. 2022). However, in this study, *B. grandis*, *B. handelii*, and *B. henryi* also share a common ancestor with the *B. pedatifida*, *B. jinyunensis*, and *B. pulchrifolia* group, and they are all placed in the same clade, indicating that they are closely related. In previous studies, these species were divided into two clades: one where *B. handelii* and the *B. pedatifida*, *B. jinyunensis*, and *B. pulchrifolia* group shared a common ancestor, and another where *B. grandis* and *B. henryi* shared a different common ancestor. Therefore, *B. grandis* and *B. henryi* were more distantly related to *B. handelii* and the *B. pedatifida*, *B. jinyunensis*, and *B. pulchrifolia* group in previous phylogenetic analyses based on the ITS1, ITS2, and 5.8S gene regions. This inconsistency with previous studies suggests that further in-depth analysis is required (Feng et al. 2022).

## Discussion and conclusion

This study reports the chloroplast genome of *Begonia pedatifida* for the first time. In our study, the phylogenetic analysis based on the chloroplast genome sequences reveals that *B. pedatifida*, *B. jinyunensis*, and *B. pulchrifolia* share a common ancestor, which is consistent with previous studies using the ITS1, ITS2, and 5.8S gene regions. However, an unexpected result is that *B. grandis*, *B. handelii*, and *B. henryi* also group with these species in the same clade, indicating a closer relationship among them than previously thought. Earlier phylogenetic studies had placed *B. grandis* and *B. henryi* in a separate clade, suggesting they were more distantly related to *B. handelii* and the *B. pedatifida* group (Feng et al. 2022). This discrepancy highlights the need for further investigation, as it could be attributed to the different genetic regions analyzed, with the chloroplast genome providing a more comprehensive insight into the evolutionary relationships within the genus. Given the limited availability of chloroplast genome sequences in public databases like NCBI, expanding genomic data will be crucial for clarifying these relationships. Furthermore, there has been little research on the chloroplast genomes of the *Begonia* genus. Therefore, further research is needed to elucidate the phylogenetic position of *B. pedatifida*. This study provides genetic resource information for future studies on *B. pedatifida.*

## Supplementary Material

Figure S3.jpg

Supplemental Material.docx

## Data Availability

The complete chloroplast genome sequence of *B. pedatifida* has been submitted to the GenBank database. The accession number for this sequence is OR288087.1, which is automatically generated by the NCBI. In addition, the associated BioProject number is PRJNA1097669, the SRA accession number is SRR28591265, and the Bio-Sample number is SAMN40876394.
