# Supplementary figures and images for: The complete chloroplast genome sequence of Begonia pedatifida
Source: Mitochondrial DNA B Resour. 2024 Sep 30;9(10):1302–6. doi: 10.1080/23802359.2024.2410444 (PMC11445898; doi:10.1080/23802359.2024.2410444)

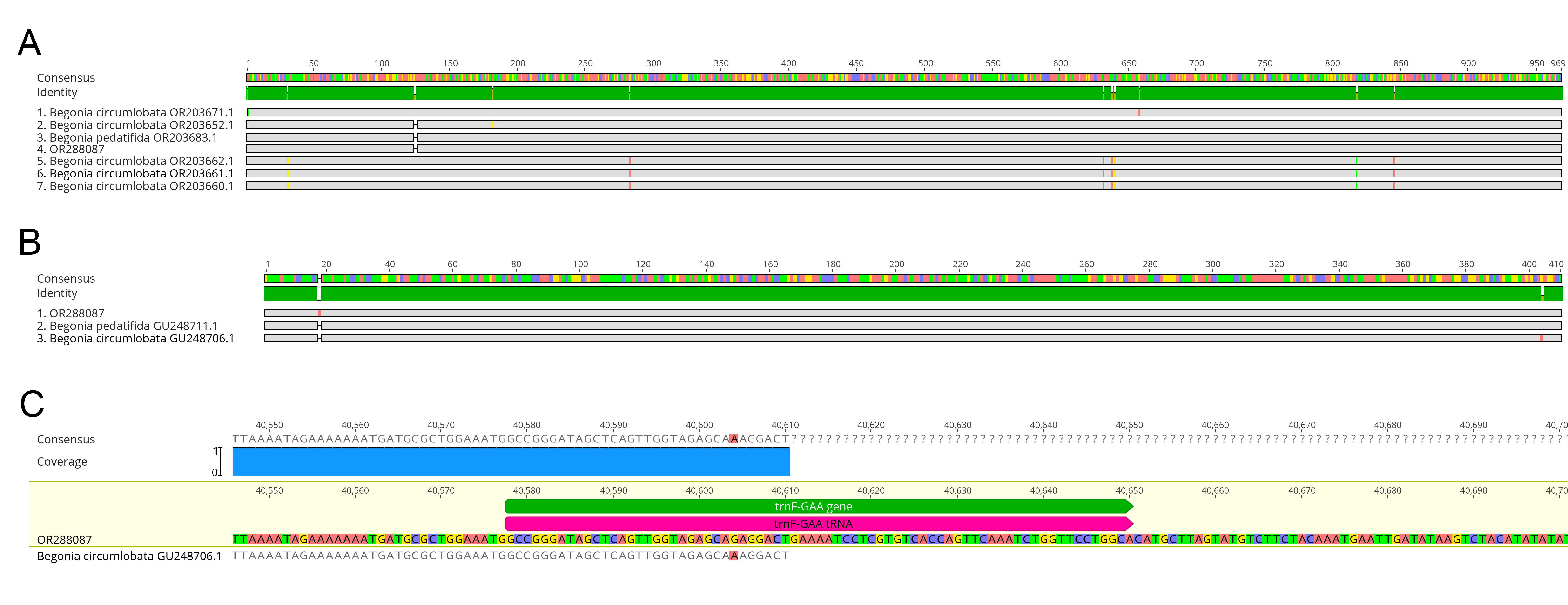

Supplement: Figure S3.jpg [file TMDN_A_2410444_SM2668.jpg]
