# Supplementary material for: The complete chloroplast genome sequence of Begonia pedatifida
Source: Mitochondrial DNA B Resour. 2024 Sep 30;9(10):1302–6. doi: 10.1080/23802359.2024.2410444 (PMC11445898; doi:10.1080/23802359.2024.2410444)
Supplement: Supplemental Material.docx [file TMDN_A_2410444_SM2667.docx]

**Supplemental Material**


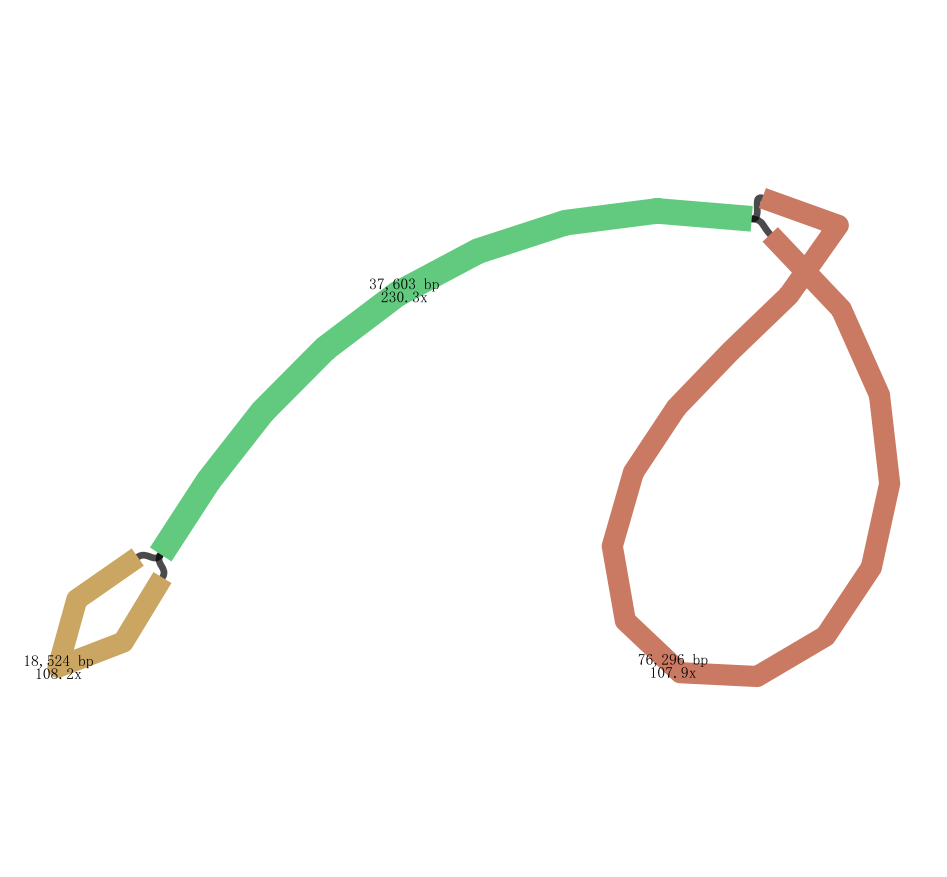


**Figure S1.** The schematic representation of the coverage depth for the entire chloroplast genome of *Begonia pedatifida* using Bandage. The numbers indicate the depths of different regions.


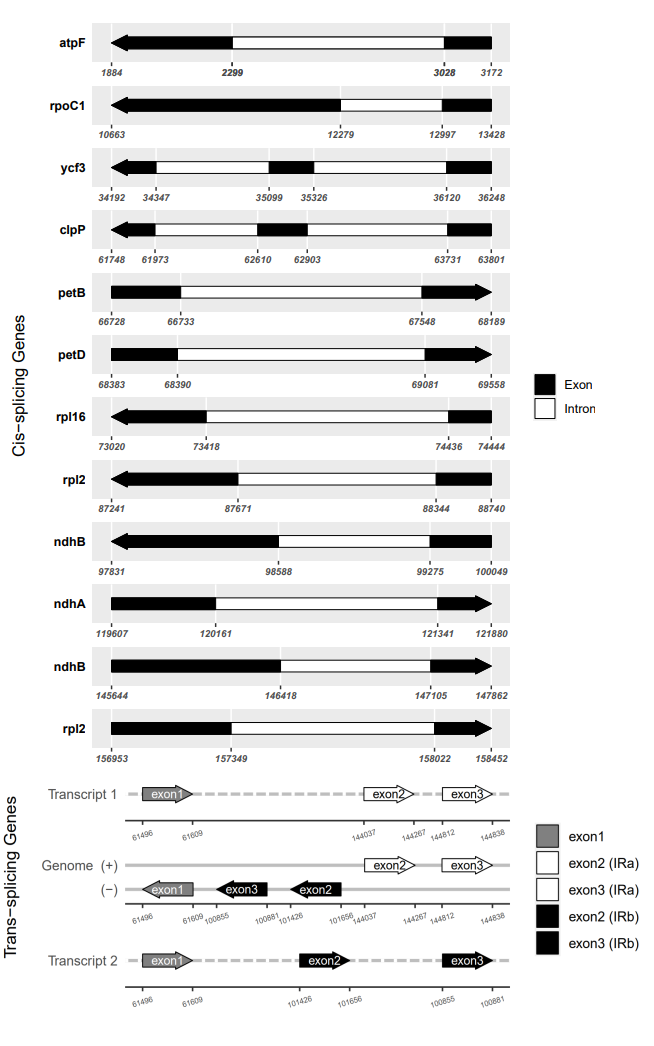


**Figure S2.** Schematic map of the cis-splicing genes and trans-splicing gene *rps12* in the chloroplast genome of *Begonia pedatifida* using CPGView. The exons of the cis-splicing genes are shown in black; the introns are shown in white. The arrow indicates the sense direction of the gene. Please note that lengths of exons and introns are not drawn to scale.


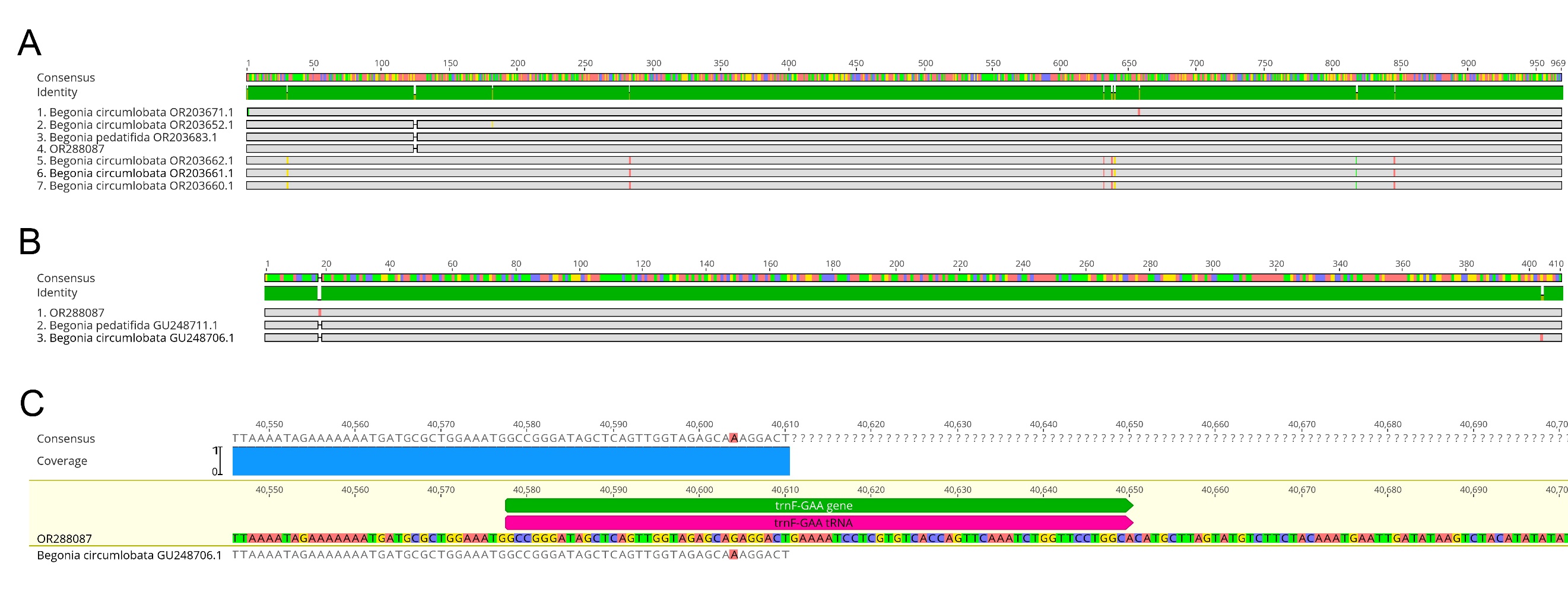


**Figure S3.** Molecular identification and comparison of *Begonia pedatifida*, *Begonia circumlobata*, and the collected specimen using DNA barcoding sequences. **A.** Multiple sequence alignment of rpl16 coding sequences. All available rpl16 CDS sequences for *Begonia circumlobata* and *Begonia pedatifida* from NCBI were included. OR288087 represents our collected specimen. The alignment demonstrates 100% sequence identity between OR288087 and *Begonia pedatifida*, confirming the taxonomic identity of our sample. **B.** Multiple sequence alignment of trnL-trnF intergenic spacer regions. Distinct sequence characteristics are observed among the three taxa. Notably, OR288087 exhibits a unique insertion of sequence 'A' around the 20 bp position, while *Begonia circumlobata* shows a G to A substitution between positions 400-410. **C.** Mapping of *Begonia circumlobata* trnL-trnF sequence to the OR288087 chloroplast genome. The G to A mutation in *Begonia circumlobata*'s trnL-trnF sequence (positions 400-410) corresponds to a substitution within the trnF coding region, a relatively conserved locus. This observation suggests a closer phylogenetic relationship between OR288087 and *Begonia pedatifida*. In conclusion, based on the molecular evidence presented in panels **A**-**C**, we identify our collected specimen (OR288087) as *Begonia pedatifida*.


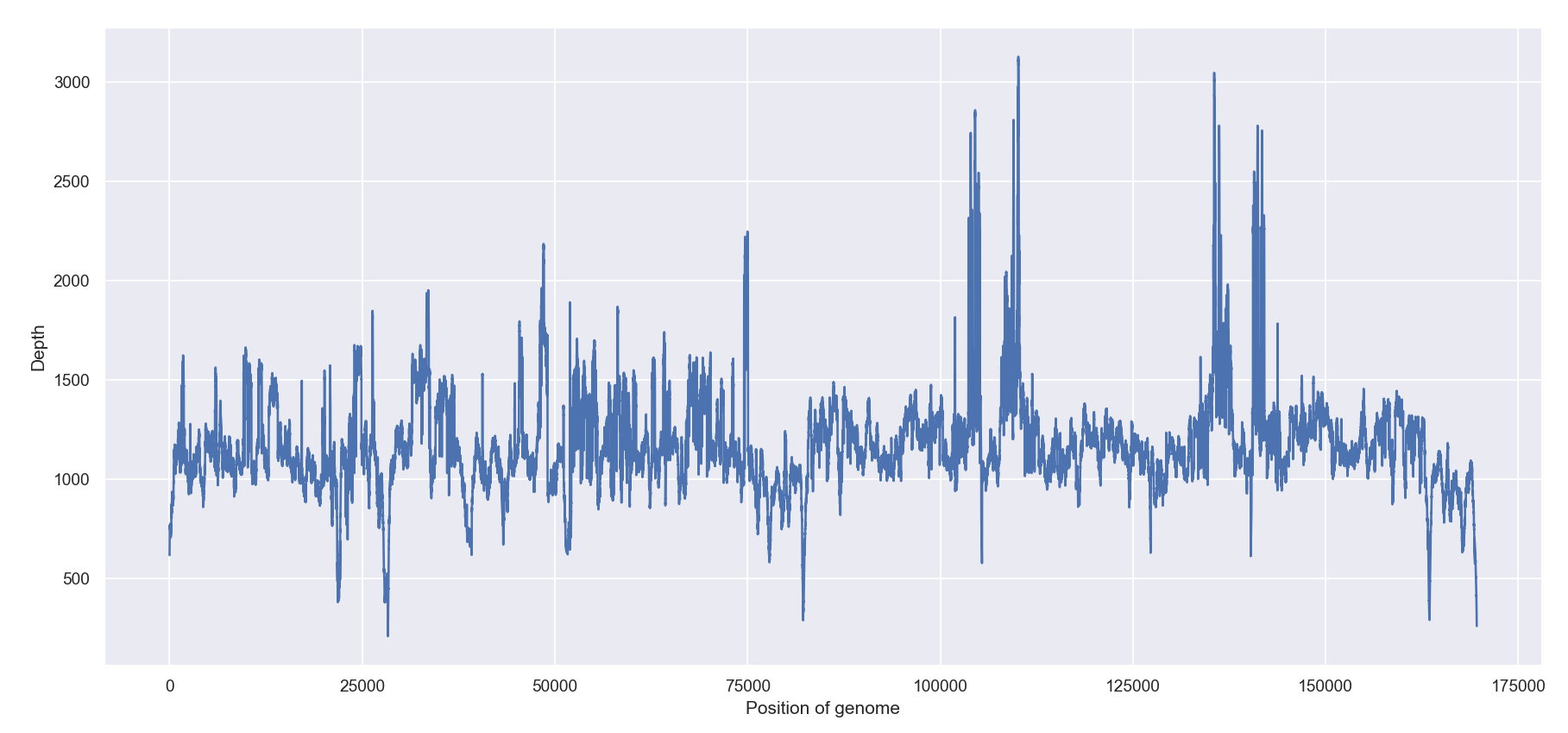


**Figure S4.** Chloroplast genome sequencing depth distribution for *Begonia pedatifida*. The graph illustrates the chloroplast genome sequencing depth distribution for species *Elaeocarpus duclouxii*, with the horizontal axis representing genomic position and the vertical axis indicating sequencing depth.
